# Natural Melanin: Current Trends, and Future Approaches, with Especial Reference to Microbial Source

**DOI:** 10.3390/polym14071339

**Published:** 2022-03-25

**Authors:** Noura El-Ahmady El-Naggar, WesamEldin I. A. Saber

**Affiliations:** 1 Department of Bioprocess Development, Genetic Engineering and Biotechnology Research Institute, City of Scientific Research and Technological Applications (SRTA-City), Alexandria 21934, Egypt; 2 Microbial Activity Unit, Microbiology Department, Soils, Water and Environment Research Institute, Agricultural Research Center, Giza 12619, Egypt

**Keywords:** microbial melanin, optimization, fermentation, detection, biosynthesis, artificial intelligence, recombinant microbes

## Abstract

Melanin is a universal natural dark polymeric pigment, arising in microorganisms, animals, and plants. There is a couple of pieces of literature on melanin, each focusing on a different issue, the goal of the present review is to focus on microbial melanin. It has numerous benefits with very few drawbacks. The current situation and expected trends are discussed. Intriguing, numerous studies have provoked a serious necessity for a comprehensive assessment of microbial melanin pigments. So that, such review would help scholars from diverse backgrounds to realize the importance of melanin pigments isolated from microorganisms, with this aim in mind, information, and hypothesis from this review could be the paradigm for studies on melanin in the next era.

## 1. Introduction

The natural pigment, melanin, is abundantly detected in a wide array of higher organisms (animals, and plants), and microorganisms (fungi and bacteria), as well, where such pigments play vital and multi-function roles. They are generated by the oxidative polymerization of phenolic or indolic molecules [1]. Physically, melanin is amorphous with a dark brown to black, as the predominant color, sometimes the reddish, and yellowish colors have also been observed. In humans, melanin determines skin color [2,3].

Chemically, they have a high molecular weight with a negatively charged hydrophobic nature, forming complex polymers that resist the concentrated acids, light, and reducers, what is more, they are very thermostable, some can resist thermolysis up to 600 °C [1,4]. But, on the other side, susceptible to oxidizing agents (bleaching processes), and soluble in both alkali and phenols [5].

Collectively, the physicochemical features of melanin allow them to carry out multitasks, as a result, melanin has potential multi-applications in a variety of biological, environmental, and technological fields, for instance, as antitumor, scavengers of free radicals, antimicrobial, neuroprotector, synthesizer of nanoparticles, remediator of radioactive residuals, antivenin stimulator, liver protectant, anti-inflammatory, and protector of the digestive system [3,6,7,8,9,10,11]. Therefore, melanogenesis is a vital process for living organisms.

Although melanin protects the pigmented cells and tissues by adsorbing potentially harmful substances (drug and chemicals) in pigmented tissues, this process may represent a drawback, since long-term exposure, may accumulate high levels of chemicals, which ultimately may cause degeneration in the melanin-containing cells, and secondary lesions in surrounding tissues [12].

Owing to the increasing demand for the melanin pigment, conducting studies on the production of melanin and selection of new unordinary sources are a must. Fortunately, the microorganism can efficiently perform the task. Microbial melanin proved its substantial importance in various fields. Several modern approaches are expected to share extensively in the next scenarios in microbial melanin production e.g., application of the statistical approach and artificial intelligence [13], using economy substrate [14], recombinant microbes and/or random mutagenesis [15], and green nano-melanin’s synthesis approach [16].

**Figure 1:**
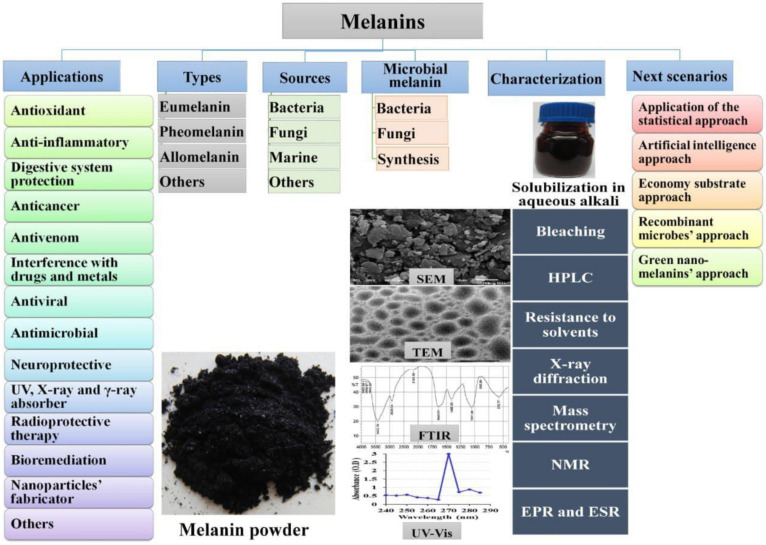
Diagrammatic scheme of the various topics covered by the current review.

The current review is spotting the light on natural melanin with special referencing to the microbial source. The future perspectives of such life-keeper pigments were highlighted. The overall structure of the current review was organized as introduced in Figure 1.

## 2. Applications of Melanin

Melanin has occupied a unique position in various biotechnological, medicinal, and environmental aspects, this may be due to the variability in biological features, physicochemical properties, chemical structure, making them have a widespread application in everyday life.

### 2.1. Free Radical Scavenger

**Figure 2:**
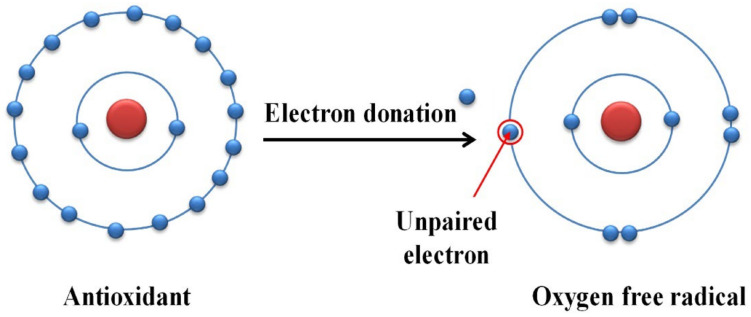
Mode of action of melanin as an antioxidant.

Melanin is reported to have a vital role as an antioxidant by neutralizing the free radicals that cause stress on many parts of the body. Melanin is well known for its antioxidant property by reducing the generation of reactive oxygen species [17]. Melanin stabilizes the free radical damage by acting as an acceptor or doner of an electron to make it stable before it can oxidize other cell components (Figure 2). The purified melanin pigment produced by bacteria e.g., *Streptomyces glaucescens* exhibited good scavenging activity [4,8], acting as an antioxidant with valuable applications in the pharmaceutical fields.

### 2.2. Anti-Inflammatory Activity

The anti-inflammatory behavior of microbial melanin was reported [9]. Inflammation is the body’s natural response induced by tissue injury or infection and interrelates to antioxidation. Melanin is well known for its antioxidant property, which can minimize the generation of the reactive oxygen species and thereby help in reducing the inflammatory response. The topical application of melanin against formalin-induced rat-paw edema reported a strong anti-inflammatory action of melanin and thus can be a potential anti-inflammatory medication [10]. Melanin can inhibit the activity of cyclooxygenase (COX), Lipoxygenase (LOX) enzymes, which have significant roles in the regulation of inflammatory responses. COX is a limiting enzyme in inflammation as it is involved in the conversion of arachidonic acid to prostaglandins, which are associated with many inflammatory diseases. LOX produces leukotrienes, thus is important in the pathophysiology of inflammatory diseases. Another antioxidant activity is accompanied by a reduction of inflammation as both these properties are interrelated to each other [17].

### 2.3. Digestive System Protection

Lipid peroxidation is the main cause of cell damage, through the oxidative degradation of lipids, free radicals take electrons from the lipids in cell membranes, thus cell destruction occurs. The lipid peroxidation inhibition ability of melanin was reported. For example, inhibition of eye lipid peroxidation in the eye by melanin effectively prevented uveitis [10]. When exposed to free radicals, lipid peroxidation of liver cells was inhibited by melanin extracted obtained from black tea [11]. Additionally, melanin effectively hinders lipid peroxidation of liver microsomal membranes, and further protects hepatic peroxidation that occurs in adjuvant-induced disease in rats, the observed effect was activation by melanin. Another hepatoprotective effect of melanin was stated against hydrazine-induced liver injury since melanin plays a vital role during liver intoxication by heavy metals through preventing the development of liver intoxication and improving liver functions as well [10,11]. 

Further beneficial gastrointestinal health of melanin was mentioned. Previous studies on gastric ulcers revealed that melanin constrained the secretion of gastric juice and thus prevent ulceration in gastric mucosa in rats, another important replenishment role, melanin can drive the renewal of the mucus levels in ethanol-depleted gastric cell walls, and powerfully defends against ulcers induced by alcohol, indomethacin, stress or the combined ulcerogenic activity of both aspirin, and stress [10,12].

### 2.4. Anti-Cancer Activity

During cancer-curing, melanin is used as a cancer remedy, also to protect patients undergoing radiation doses against the harmful effects of gamma rays [4,18]. In vitro, melanin exhibited potent cytotoxic activity against HFB4 skin cancer cell line was reported with an additional benefit of little cytotoxicity towards normal non-cancerous cells, supporting that melanin pigments can be applied as promise anti-malignant tumor [4]. However, the cytotoxicity of black melanin was concentration-dependent. In comparison to Doxorubicin (4.05 μg), melanin inhibited the growth of the cell lines HEPG-2, HCT-116, and MCF-7 with IC_50_ values of 6.15, 5.54, and 7.91 μg, respectively [19]. In-vitro, 60 μg melanin recorded 53% inhibition of the hepatocellular carcinoma cell line [18]. 

### 2.5. Melanin as Antivenom

Melanin demonstrated a neutralization effect against several venoms. A study performed on model animals injected immediately, in the same place of venom, with melanin after the venom administration (3 mg per mouse). The greatest antivenom impact was discovered against Japanese mamushi snake venom. Melanin’s low venomousness, along with its antagonistic action against various venoms, may enable successful life-saving therapy for snakebites [11]. 

### 2.6. Interference with Drugs and Metals

Melanin may perform a task in protecting against antimicrobial drugs, therefore, in humans, the lack of melanin leads to several abnormalities and diseases [3,12]. Broad pharmacological and physicochemical investigations concentrate on the inter-relationships of melanin with metals and medications, which promptly link to melanin and are held in pigmented tissues for significantly longer periods, in this way, melanin may be used as a vehicle or channel for drug delivery, acting as a drug carrier or transporter to the site of action, especially through interacting with orally administered drugs [10]. For example, iron-deficiency anemia was treated using melanin-iron complex, this led to a significant reduction in symptoms, greater bioavailability of iron, and rarer side impacts than the treatment with standard drugs does, signifying that melanin-iron complexes might assist in improving hematopoietic performance and could be exploited as a safe, efficient new iron tonic [20].

Another, melanin’s pigmented cells and their adjacent tissues were found to be protected from harmful substances, where melanin can absorb the harmful substances, preventing or neutralizing the potential damage that occurred by such substances. The physiological role of the metallic-binding feature of melanin is not clear, but possibly melanin gradually released the binding harmful substances in nontoxic concentrations [10].

In the biomedical field, the human antigenic response is among the main obstacles usually linked to the used biomaterials, along with the lack of stability under certain physiological conditions. This challenge had been overcome in the case of melanin due to the lack of enzymes that are capable of decomposing melanin, as well as biostability and a lesser likelihood of long-term accumulation in organs, which is normally linked with various harmful effects [9,16].

### 2.7. Antiviral Feature

The replication of human immune-deficiency viruses (HIV) can be inhibited by the synthetic soluble melanin, furthermore, the selective antiviral activity of synthetic soluble melanin against SARS-CoV2 and HIV was revealed without toxicity to the host cells [17]. Melanin complexes also exert various favorable effects on both in vitro and in vivo animal models, these melanin complexes could serve as a supply of biopolymers for the creation of new medications with wide applications in infectious viral diseases [21]. A pioneering study proved that melanin precursors like 5,6-dihydroxyindole-2-carboxylic acid and l-3,4-dihydroxyphenylalanine strongly interact with the spike protein of the SARS-CoV2, confirming that melanin and its precursors could be utilized as effective antiviral compounds [22].

### 2.8. Antimicrobial Action

Crude melanin pigment can usefully manage microbial diseases [23]. For instance, melanin of *Streptomyces* sp. had proved antibacterial behavior against *Lactobacillus vulgaris,* and *Escherichia coli* [24]. Melanin complexes were reported to have the same antimicrobial effects on pure cultures of *Candida albicans,* and *Helicobacter pylori* [21], suggesting the success of melanin-based therapy as anti-microbial pathology.

### 2.9. Neuroprotective Agent

Melanin promotes chemical transport, resulting in faster nerve impulses and further acting as neuroprotection [25]. These particular features of melanin elucidate their incidence in organs, and tissues that are connected with impulse transmission systems, like body skin, nervous system, and retina [26]. Therefore, melanin metabolism abnormalities may have a role in the etiology (genesis) of illnesses such as Parkinsonism, which was shown to be associated with melanin metabolism disorders. Studies on the post-mortem brains of Parkinson’s disease patients, animal models, and in vitro cultures indicate the presence of programmed cell death (apoptosis) in the substantia nigra region, which was confirmed through DNA fragmentation and typical morphological changes in the brain’s cell [27].

After induced destruction, bacterial melanin supports the survival of neurons in the substantia nigra pars compacta and preserves dopaminergic neurons, suppressing secondary inflammation in damaged brain tissue. Low concentrations of bacterial melanin are non-toxic and do not produce side effects. Moreover, bacterial melanin has been evaluated as a strong activator of regeneration and motion recovery in rat models subjected to unilateral depletion of the substantia nigra pars compacta. It stimulates the healing of a damaged motion tract and the regeneration of a damaged peripheral nerve fiber, produces capillary dilatation in the lesion area, and so improves blood flow in brain tissue [25,28]. Another protective mechanism of neuromelanin pigments is that they attach and sequester the toxic metals to form stable complexes that prevent neuronal toxicity [27]. Another study confirmed that the bacterial melanin has been used as a neuroprotector. It aids in the recovery and regeneration processes following central nervous system lesions. The role of bacterial melanin after the destruction of substantia nigra is of great interest. The firing rate of substantia nigra pars compacta dopaminergic neurons is significantly increased by bacterial melanin. An increase in the firing rate of substantia nigra neurons can aid in the recovery of the substantia nigra after neuronal degeneration [29].

### 2.10. UV X-, and γ-rays Protective

Melanin is used in pharmaceuticals and cosmetics, especially in photoprotective-based creams that are utilized to protect against ultraviolet (UV) radiation [30], X-ray, and γ-ray [31], particularly in optical lenses as eye protectants [22].

Almost all living creatures are exposed to UV radiation that spreads on the earth’s surface regularly. The UV spectrum (between 200 and 400 nm) is generally split into three regions; UV-A (320–400 nanometers), UV-B (280–320 nanometers), and UV-C (280–200 nm). The potential damage of UV radiation increases exponentially with decreasing wavelength. Sunburn, premature skin photoaging, skin malignancy, and immune system suppression have been related to UV radiation exposure [8]. 

Melanin in the skin does not entirely prevent UV from accessing and harming cells; rather, it decreases UV transmission to the nuclei. Consequently, studies are being conducted to give enough UV protection against the development of skin cancer. Sunscreen products, unlike melanin, frequently utilize a mixture of chemical filters, which raises the risk of adverse effects, melanin, on the other hand, is considered superior UV-sunscreens as a component of photoprotective creams since their maximal absorption is often within the UV spectrum [8].

### 2.11. Radioprotective Therapy

Oral melanin-based products are used for the protection of people who expose to radiation. The oral-radioprotective efficacy of melanin is used as edible or drinkable for alleviating and/or preventing one or more side effects associated with radiation exposure. In a study on mice, one-hour post-eumelanin feeding (15 mg/kg body weight), the mice were subjected to a total body irradiation dose of 9 Gy, the defensive action of melanin was due to hindering the radiation-induced hematopoietic harms. Melanin prevents apoptosis in splenic tissue, diminishes oxidative stress in hepatic tissue, and abrogates immune imbalance [32,33].

During cancer treatment, radiation therapy can damage the normal cells. Therefore, internal administration by the radioprotective materials is applied to shield normal organs without protecting the cancer tissue, leading to enhancement of the effectiveness of radiation treatment by permitting higher tumoricidal dosages. In this case, melanin protects against the harmful effects of gamma rays by preventing the formation and/or scavenging the free radicals, which instigate DNA damage [32].

Novel melanin nano-shells, used as radioprotector approach, were reported, attempting to apply the melanin coated silica nanoparticles for protection of bone marrow from ionizing radiation during cancer radiation therapy, implying a thinkable future medical implication of melanin for radioprotection in humans [34]. Melanin nano-shells are also utilized to defend against radiation. It can be made in or on clothes, safety gear, or packaging material, for example. The material can be incorporated into the wall of a room, ceiling, floor, building, vehicle, aircraft, ship, spaceship, and submarine [32].

### 2.12. Bioremediation of Radioactive Residuals

There is considerable worry about the health dangers connected with the extensive development of nuclear projects in energy generation, medicine, agriculture, and industry. Bacterial melanin was utilized to immobilize uranium in uranium-polluted soils. For fostering such in-situ procedure of uranium bio-immobilization, the one-time addition of tyrosine is applied to soil, This, in turn, exploits the ability of native microbes to produce melanin, resulting in uranium immobilization. Consequently, melanin-delivering microorganisms can be employed in the bioremediation of radioactive waste like uranium [35].

Species of melanized fungi is another group of melanin producers that have been found to thrive in regions of high-radiation capacity as Chernobyl, and in cooling pool water of nuclear reactors, similarly, numerous laboratory investigations have shown that melanized fungi are tolerant to different levels of UV and ionizing radiation [32]. It was determined that melanin’s radioprotective effect in microorganisms was owed to a mixture of physical shielding, and physiological activities that quenching the harmful nature of free radicals [33,35].

### 2.13. Nanoparticles’ Fabricator

The biopolymer of melanin might function as a reducing, and stabilizing mediator in the production of silver and gold nanostructures. Silver nitrate and chloroauric acid were converted to silver and gold nanostructures by the L-DOPA-melanin pigment, respectively. The silver nanoparticles were shown to have strong antifungal activities and further may be used as efficient paint additives [36].

### 2.14. Food Industry

The microbial pigment, melanin has received considerable attention because of its useful biological activities in food. Melanin is used as a food colorant and nutritional supplements as natural ingredients, replacing the chemically synthesized pigments which cause harmful effects in the natural environment [37,38]. In this respect, melanin pigment produced by sponge-associated actinobacterium Nocardiopsis alba was used for the environmentally benign synthesis of silver nanostructures, which was useful for food packaging materials [39]. The characteristics of the broad spectrum of activity against food pathogens of silver nanostructures give an insight into their potential applicability in the incorporation of food packaging materials and antimicrobials for stored fruits and foods [8].

### 2.15. Other Uses

Other biological, environmental, and technological applications for melanin pigments have been mentioned. Melanogenesis plays a vital role in many clusters of free-living organisms to protect them from ecological stress conditions that improve the survival and competitiveness of the organisms [15]. In plants, for example, melanin is incorporated in the cell walls as strengtheners [40].

One of the most common features of melanin pigments is the capacity of eumelanin and pheomelanin to bind different metal ions [41]. Ecologically, fungal melanin showed potential metal-ions chelating ability [10]. Melanin is an extremely effective, and fast ion exchange molecule that binds toxins, chemicals, and heavy metals, serving as a radical sink [5]. This feature is important biologically because it allows melanin to chelate substances and control their entrance into cells [42]. These pigments are thought to act as a reservoir or trap for metal ions, such as Ca (II), Zn (II), Cu (II), and Fe (III) [43]. 

Another detoxification role was reported by microbial melanin, by which mycotoxin secretion was inhibited [9]. In agriculture, microbial melanin has the potential to be used as bioinsecticides [44]. Finally, melanin has various industrial applications such as bioplastics, paints, and varnishes [45], as well as, in bioelectronics, melanin could be useful as semiconductors [46].

## 3. Types of Melanin

**Figure 3:**
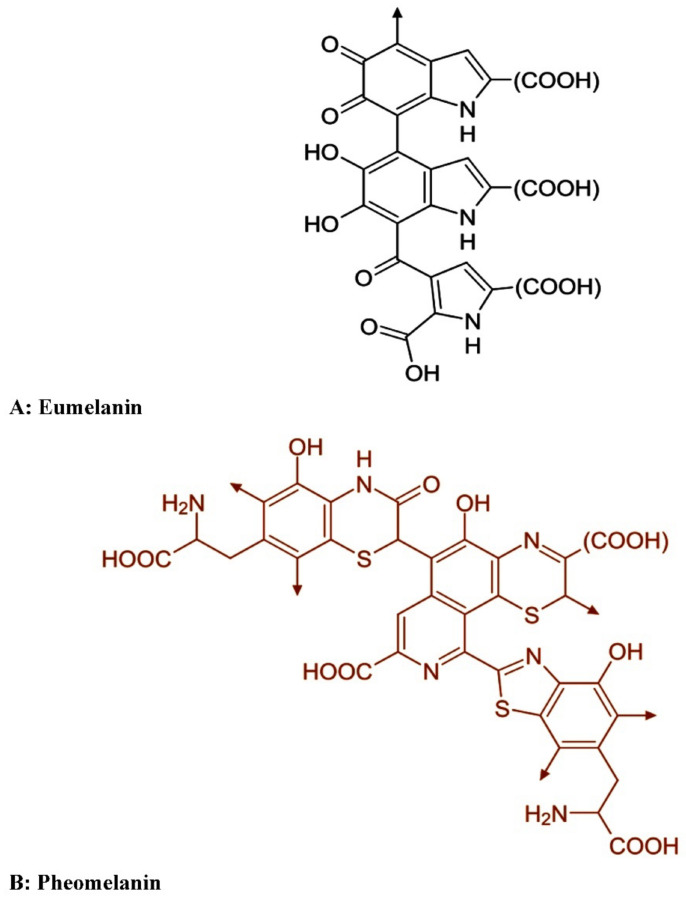
Part of the structural formula of the most common types of melanins; eumelanin (**A**), and pheomelanin (**B**).

Because melanin polymer is an assemblage of smaller molecules, the quantities and bonding patterns of these molecules influence the kind of melanin to a considerable extent. The main four kinds of melanin were identified based on color and structural classifications i.e., eumelanin, pheomelanin, allomelanin, and others that were occasionally created (Figure 3).

### 3.1. Eumelanin

Eumelanin is mostly black to dark brown pigments that are formed in human hair and skin and can also be generated by some bacteria and fungi [47]. Eumelanin is created by the oxidative polymerization of tyrosine and/or phenylalanine into L-3,4-dihydroxyphenylalanine (L-DOPA), which is subsequently transformed into dopachrome and finally melanin [48,49].

### 3.2. Pheomelanin

Pheomelanin is a red or yellow pigment found, mainly, in red human hair that is originally formed similarly to eumelanin, but L-DOPA undergoes integration of cysteine in the polymer (cysteinylation), and therefore, pheomelanin contain sulfur [15].

### 3.3. Allomelanin

Allomelanin is a heterogeneous pigment found in many fungi and plants that feature a nitrogen-free heterogeneous category of polymers. They are derived from many sources, including dihydrofolate, homogentisic acid, catechols, and others [4,9]. This type includes melanin, formed from 1,8-dihydroxy naphthalene (DHN) compounds, and water-soluble pyomelanin created when homogentisic acid, a by-product of the tyrosine breakdown pathway, accumulates and polymerizes [50].

### 3.4. Other Types

Other less frequent melanin are occasionally created as a result of a divergence from the preceding melanin types. Trichochrome pigments (previously known as trichosiderins) are formed in the same metabolic route as eumelanin and pheomelanin, by having a lower molecular weight than the original melanin molecules [51]. Neuromelanin is dark insoluble pigments generated in a distinct region of catecholaminergic neurons in the brain. Neuromelanin granules should have a mixture of pheomelanin in the core and eumelanin in the surface [52]. Humans contain the most neuromelanin, which is present in smaller levels in several other non-human primates but is completely lacking in many lower species’ brains [53]. Human neuromelanin has been demonstrated to bind transition metals like iron as well as other potentially hazardous compounds. As a result, it may perform critical roles in apoptosis, neurodegeneration, and Parkinson’s disease [54]. Individuals with Parkinson’s disease had half the quantity of neuromelanin in the substantia nigra as compared with the patients of the same age who did not have Parkinson’s. The concentration of neuromelanin increases with age, implying a function in neuroprotection [53,54].

## 4. Source of Melanin

The biological supply of melanin is miscellaneous. Melanin can be found in a variety of biological and synthetic forms. The majority of which are available in a commercial form for purchase. Synthetic melanin polymers are generated by auto-oxidation and polymerization of phenolic or indolic compounds (e.g., catechols). During the process, melanin absorption spectra raise monotonically from 700 to 250 nm [55]. Another route, by the catalytic action of tyrosinase on tyrosine or 3,4-dihydroxyphenylalanine [40].

The largest producers, however, are marine cephalopods, this is the best-studied melanin so far, which is expelled out by Sepia as a defense strategy against their adversaries [56]. Plants are another source, in which the vegetable melanin are obtained by a chemically treated process, whereby a vegetable crude material-containing polymers or monomeric units of the flavonoids is processed to extract melanin [40].

Another key source of melanin is microorganisms (bacteria and fungus). Microbes’ ability to synthesize melanin is largely determined by their virulence to host associations and their ability to protect themselves from environmental stresses such as temperature extremes, ultraviolet rays, and solar radiation, as well as adverse chemical stress conditions, e.g., oxidant-mediated damage, lytic-enzymes, heavy metal toxicity, and antimicrobial medications [9,57].

## 5. Why Microbial Melanin?

The synthetic melanin polymers cannot be compared with natural melanin from the environmental point of view, and the quality, as well. The latter is safe and cost-effective than the artificial one, especially if the proposed scenarios in the current review are considered. However, microorganisms have recently gained popularity as an alternative to chemical melanin production. Therefore, several attempts have been done to obtain microbial strains qualified to synthesize a high quantity of melanin pigment for several reasons. Melanin obtained from microbes has great advantages over synthetic, and those from animals and plants melanin. 

In contrast to the other biological-based methods (marine and vegetable melanin), microorganisms do not get influenced by the problems of seasonal fluctuation during the production progression and they can modify their mechanisms according to the medium composition and growth conditions provided [58]. Meaning that the biosynthesis of melanin pigments by microbes is independent of meteorological conditions that interfere with the production process. The ease and speed of growth with no detrimental consequences and their ability to grow on low-cost substrates are other merits [4,57,58]. 

Another important merit of microbial melanin is the diverse microbial sources. Several reports stated various bacterial and fungal melanin, have various characteristics, and hence various applications. Under laboratory conditions, microbial species with melanogenic capacity have been examined and/or optimized for developing production processes (Table 1). For instance, *Aeromonas media* and *Pseudomonas maltophilia* were found to produce authentic 3,4-dihydroxyphenylalanine melanin pigment. Although melanin from both bacteria shares many biophysical properties, the yield of Aeromonas media was significantly higher and showed to be more effective in the protection of a bioinsecticide against ultraviolet or solar radiation than melanin of *Pseudomonas maltophilia* [44]. Another study on Actinomycetes described a newly isolated strain, *Streptomyces glaucescens* NEAE-H, capable of producing a high amount of black melanin pigment on an optimized medium of peptone-yeast extract iron agar. The in vitro trial showed anticancer activity of melanin against skin cancer cell lines [4]. 

For another group of microorganisms, the pathogenic *Cryptococcus neoformans* was able to accumulate black melanin pigments, in a medium containing phenolic substrates such as L-dopa. Pigmented and non-pigmented mutants of *Cryptococcus neoformans* cells were studied with transmission electron microscopy and electron spin resonance, both revealed a stable free-radical population in pigmented cells. The melanin deficient was less virulent for mice, and the melanized cells were also more resistant to antibody-mediated phagocytosis and the antifungal effects of murine macrophages than non-melanized cells, concluding that melanin pigment production is associated with microbial virulence by protecting cells against attack by immune effector cells [59].

**Figure 4:**
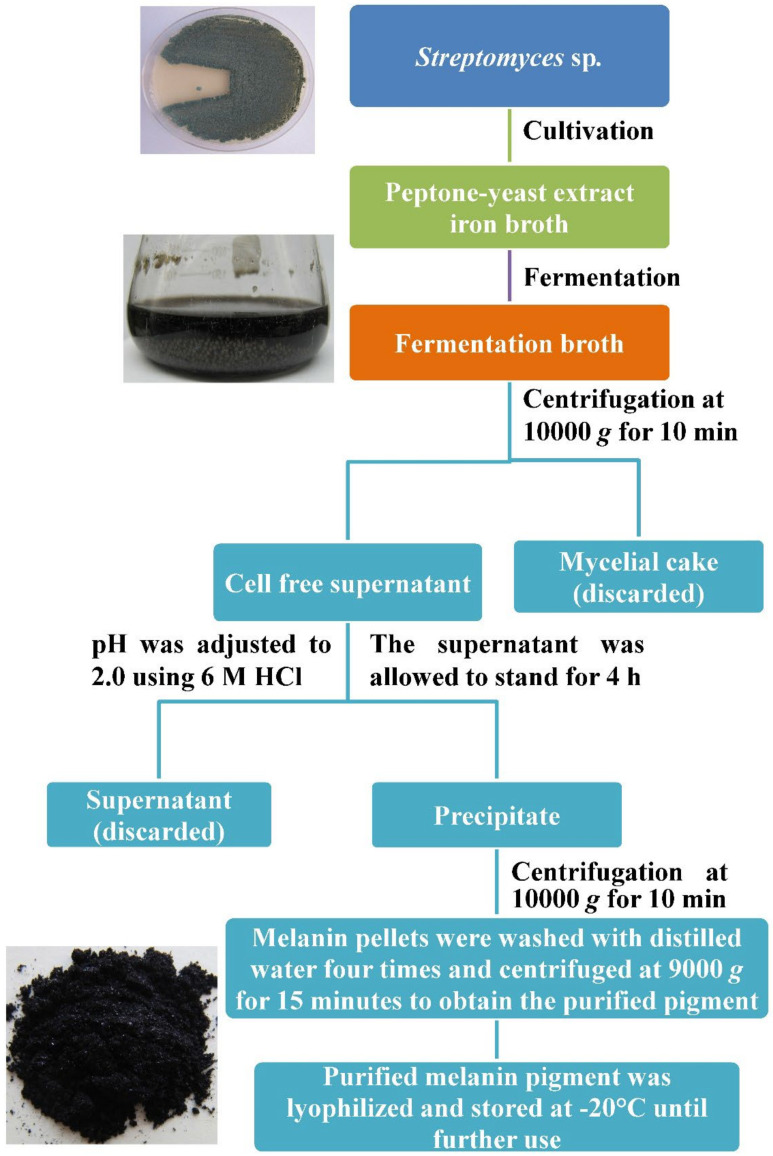
The general procedure of microbial melanin production. The graph was designed based on the data extracted from a previous study.

During an extensive search to find out cost-effective alternative sources to the conventional sources of melanin production, 102 fungal isolates were tested, and only *Amorphotheca resinae* was chosen as a promising melanin producer on peptone yeast extract glucose broth. *Amorphotheca resinae* produced the melanin rapidly, reaching up to 4.5 g/L within 14 days. Structural characterization of the purified fungal melanin indicates that it is like the eumelanin and has a high antioxidant activity because of free radical scavenging assays, suggesting a promising fungal candidate for scalable production of industrially applicable melanin [60]. However, the general procedure of microbial melanin production is introduced in Figure 4.

### 5.1. Bacterial Melanin

The synthesis of black pigments in bacteria was realized long-time ago, since then several bacteria are reported to produce diverse groups of melanin through specialized pathways or using enzymatic imbalances in modified metabolic channels [9]. The regulation of the melanin synthesis mechanism in bacteria includes transcriptional and metabolic regulation, on the other side, several biosynthesis pathways remain still mostly unspecified [1]. However, the biosynthesis of melanin exists in Gram-positive and Gram-negative bacteria, such as *Streptomyces griseus* [79], *Bacillus licheniformis* [19], *Ralstonia solanacearum* [80]. In particular, diverse species of *Streptomyces* produce melanin that is thought to be a useful criterion for taxonomical investigations [47]. The physiological growth attributes of some bacteria were well described such as these of thermo-alkaliphilic *Streptomyces* sp. That required alkaline pH (9.0), higher temperature (45 °C), and 3% of sodium chloride [9].

### 5.2. Fungal Melanin

Fungi have been stated to possess all types of melanin discovered. Melanin in fungi is considered a secondary metabolite and their presence can be naturally constitutive. Although melanin is not essential for the growth and development of fungi but can perform an extensive spectrum of biological jobs such as increased virulence in many fungi pathogenic to humans and plants [48]. In most melanized fungi the pigment is principally localized as granules or layered in fibrils in the outermost layer or embedded within the cell wall, or bound to cell wall chitin, or secreted extracellularly [8]. Several fungi were reported to secrete melanin e.g., *Aspergillus niger*, *Aspergillus nidulans*, *Alternaria alternata*, *Cladosporium carionii*, *Fonsecaea compacta*, *Exophiala jeanselmei*, *Hendersonula toruloidii*, and *Phaeoannellomyces wernickii* [48].

### 5.3. Microbial Synthesis of Melanin

The biosynthesis mechanism of microbial melanin occurs by oxidative polymerization of phenolic compounds, principally by two distinctive pathways, mainly through L-DOPA, leading to the generation of various types of melanin i.e., eumelanin, pheomelanin. A variety of important enzymes are involved in microbial melanogenesis. Melanin is often synthesized by microbes using phenoloxidases such as tyrosinases, laccases, polyketide synthase, and p-hydroxyphenylpyruvate hydroxylase. Tyrosinase (EC 1.14.18.1) is a copper protein that belongs to the polyphenol oxidases family, it catalyzes the oxidation of L-tyrosine to L-DOPA, which is then converted to dopachrome, that transformed to melanin by a sequence of non-enzymatic oxidoreduction reactions [81]. Laccases (EC 1.10.3.2) are metalloproteins with an active site containing 1–4 copper atoms. They are not linked to tyrosinases, but rather to the family of blue copper [82]. Polyketide synthases are multidomain proteins that create DHN melanin via the polyketide pathway. They are linked to animal fatty acid synthases [83]. Many microbes use these enzymes to make pigments, antibiotics, poisons, and other intermediate metabolic molecules [84]. Finally, p-hydroxyphenylpyruvate hydroxylase is a crucial enzyme in pyomelanin biosynthesis that catalyzes the creation of homogentisinic acid. This enzyme is found in many living species and belongs to the phenylalanine and tyrosine degradation pathways [8].

Microbial melanin is generated through the metabolites resulting from the two main pathways. The most widespread pathway involves melanin precursors derived from aromatic amino acids, such as tyrosine transformations. This monohydroxylated molecule is oxidized to diphenol, (yielding dihydroxylated derivatives), in which the amino group can be conserved, giving rise to L-DOPA, or eliminated before oxidation, creating molecules like homogentisate (2,5-dihydroxyphenylacetate), or homoprotocatechuate (3,4-dihydroxyphenylacetate). These molecules are oxidized directly or by certain enzymes, giving rise to benzoquinones, or dopaquinones. The autopolymerization of such quinones leads to the formation of melanin. These synthetic pathways are mediated by copper-based enzymes (tyrosinases and laccases), resulting in the yellow-red pheomelanin, the brown-black eumelanin, and a heterogeneous group of allomelanin, including pyomelanin [1,15,45].

In addition, some microorganisms can synthesize melanin from malonyl-CoA, a process mediated by polyketide synthases [1]. This pathway was first described in *Streptomyces griseus*. The pathway includes the successive decarboxylative condensation of 5 molecules of malonyl-coenzyme A, which is managed by the homodimeric type III polyketide synthase RppA, forming 1, 3, 6, 8-tetrahydroxynaphthalene (THN). A member of the cytochrome P450 family, co-transcribed with RppA, administers the oxidative dimerization of two-THN subunits to compose hexahydroxyperylenequinone (HPQ). The autopolymerization of such unstable precursor leads to the establishment of brownish HPQ melanin [1,8,15,45].

## 6. Current Trends in Melanin Characterization

**Figure 5:**
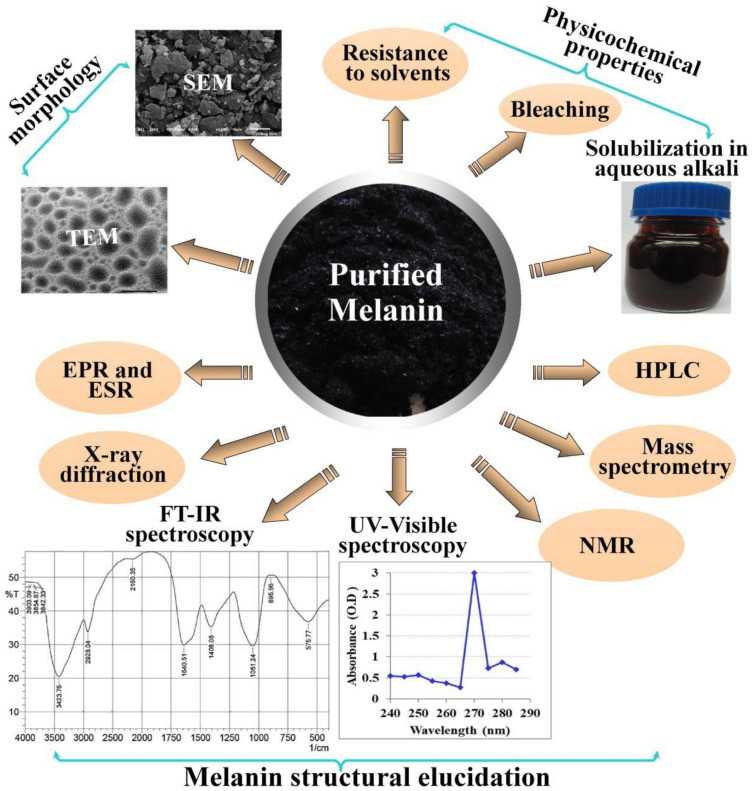
Various methods for characterization of melanin. Electron paramagnetic resonance (EPR), electron spin resonance (ESR) spectroscopy, fourier transform infrared spectroscopy (FT-IR), gas chromatography-mass spectrometry (GC-MS), high-performance liquid chromatography (HPLC), transmission electron microscopy (TEM), scanning electron microscopy (SEM), and nuclear magnetic resonance spectroscopy (NMR).

Despite their marked abundance in the global biomass, some unknown aspects about the complete chemical structure of some melanin types had to be unveiled. Because of the complex polymerization, amorphous and insoluble nature, current biochemical and biophysical practices are unable to offer a decisive chemical construction. Therefore, several identification tests of melanin have been established, including physicochemical properties (resistance to solvents, bleaching, and solubilization in aqueous alkali), surface morphology studies (scanning electron microscopy, and transmission electron microscopy), and structural elucidation i.e., electron paramagnetic resonance (EPR), electron spin resonance (ESR), X-ray photoelectron spectroscopy (XPS), fourier transform infrared spectroscopy, UV-Visible spectroscopy, nuclear magnetic resonance spectroscopy, mass spectrometry, gas chromatography-mass spectrometry, and high-performance liquid chromatography (Figure 5), these methods aid each other to introduce a complementary perspective of the melanin’s structure.

Recently, several studies and techniques were performed for the identification of melanin. Raman spectroscopy was used in the past for analyzing melanin in vivo [85]. By which, eumelanin exhibits specific Raman scattering properties that are measurable in situ, thereby providing a novel, nondestructive means for optical measurement and characterization of melanin according to their occurrence in different biological environments. 

The atomic force microscope (AFM) is a high-resolution imaging technique that implements 3D topographical data for the measurement of intermolecular forces with atomic resolution. AFM has important advantages over other microscopic methods because it provides measurements at the nanometer scale [86].

Electron spectroscopy for chemical analysis (ESCA), also known as XPS, is an elemental analysis technique, which has helped in providing the chemical natures of the nitrogen and sulfur atoms is also used to determine the quantitative atomic composition and chemistry of melanin [87].

Matrix-assisted laser desorption ionization (MALDI) analysis is a mass spectrometry coupled with fast atom bombardment, laser desorption/ionization (LDI), and matrix-assisted laser desorption/ionization. MALDI mass spectrometry was used to follow melanin degradation trends [86]. This tool proves its usefulness in analyzing natural melanin pigment, characterization of the polymer, and identification of the melanin type by detecting the monomeric units [86]. Based on MALDI, the darker a melanin, the higher its molecular mass, possibly due to a higher degree of polymerization of the molecule [87].

Due to their polymeric-like structure, melanin pigments have been extensively analyzed by pyrolysis gas chromatography coupled with mass spectrometry detection (py-GC-MS). This technique degrades macromolecules by heating them to temperatures high enough to cause bond dissociation. The pyrograms obtained are being characteristic of the original analyte. Moreover, the pyrolysate retains the original material structure information, permitting differentiation between pure analytes and a mixture, or copolymers [86].

Liquid chromatography analysis (LC-MS) can serve as a quantification method for eumelanin and pheomelanin by measuring their degradation products. The oxidation of eumelanin and pheomelanin yields specific markers, such as PTCA and pyrrole-3,5-dicarboxylic acid for eumelanin. These molecules were used to quantitatively assess the melanin in various samples, such as hair, skin tissue, fungi, melanocytes, urine, fossils, and sepia ink [86].

Other methods employed to partially characterize melanin include EPR and ESR, which show a population of stable free radicals in these molecules. Melanin polymers are known to have a paramagnetic character and o-semiquinone free radical with spin (S = 1/2). These unpaired electrons of free radicals obey the EPR effect [88].

### 6.1. Physicochemical Properties

#### 6.1.1. Resistance to Solvents

Melanin is mostly insoluble in water (cold or boiled), concentrated acids (hot or cold), and common organic solvents like methanol, ethanol, benzene, ethyl acetate, chloroform, propanol, acetone, and petroleum ether [57].

#### 6.1.2. Solubilization in Aqueous Alkali

Melanin solubility varies according to the origin, state of hydration or purity, and/or polymerization status [57]. Solubility factors also include the ionization state of the pigment’s carboxylic, phenolic and aminic groups, its polyelectrolyte characters, and its amino acid contents [89]. The melanin’s solubilization in 1 M KOH, and the precipitation detected when their alkaline solutions were acidified by 7 M HCl can be explained by the state of aggregation of melanin being affected by the pH. Lowering the pH of a melanin solution encourages the formation of aggregates and sedimentation [90] while increasing the pH causes the granules to break down into minor particles-oligomers with a poorer degree of polymerization. The presence of ionizable units and hydrophobic interactions within the molecule is responsible for this behavior [41]. Water bounds to the melanin molecule in large amounts (30% of its weight in some cases) and is likely important in maintaining its solvent-swollen state. Once the polymer is dehydrated, it becomes further aggregated and almost completely misses its capacity for physicochemical interactions [90].

#### 6.1.3. Bleaching

The bleaching of melanin solutions by oxidizing molecules such as sodium hypochlorite (NaOCl), and hydrogen peroxide (H_2_O_2_) has been related to pigment deterioration [90]. Solubilization of melanin in a strongly alkaline solution is determinate for the bleaching process, as this generates ionization of the hydroxyl phenolic units of melanin that favors reactivity with the oxidizing agent [10]. The decolorization of the pigment is mainly due to the degradation of melanin. The reaction mechanism, with H_2_O_2_, includes a nucleophilic attack of OOH^−^ ions, which induces a ring-opening reaction leading to the formation of quinone epoxides responsible for the bleaching of melanin [15,90].

### 6.2. Surface Morphology

To investigate the structure of natural and synthetic melanin, transmission and scanning electron microscopy were generally utilized. The researchers have stated that the synthetic eumelanin appears to be amorphous solids, while the natural eumelanin looks to be minute spheres [47]. Moreover, the electron microscopy investigation shows that the particles of natural melanin are non-porous spherical (around 30 nm in diameter) that tend to join as aggregates. Then these aggregates associate with loose clusters of melanin agglomerates [91].

### 6.3. Structural Elucidation

#### 6.3.1. UV-Visible Spectroscopy

A characteristic property of melanin, wavelength scanning shows a peak in the UV region, ranging from 200–700 nm of extracted *Streptomyces glaucescens* melanin pigment, and no peaks were reported out of this region, especially the visible region [4]. The highest peak absorption was perceived in the UV region at 250 nm, which then declined with the progress towards the visible area. However, the exact peak absorbance of melanin varies according to the source, and each source has maximum UV-Vis absorption peaks. For example, the purified *Chroogomphus rutilus* melanin has an extreme absorption peak at 212 nm [92], while *Streptomyces bikiniensis* M8 melanin at 230 nm [93]. Whereas wavelength scan of melanin synthesized by *Actinoalloteichus* sp. MA-32 displayed the highest absorption peak, being at 300 nm [7].

#### 6.3.2. FT-IR Spectroscopy

The FT-IR spectrum has been widely used for the characterization of fungal melanin. FT-IR is considered to be the most informative, well-resolved, and non-destructive method, providing information on functional groups such as aromatic ring CH stretching, hydroxyl group, and N–H stretch, reflecting a detailed structural analysis of melanin [57,78].

#### 6.3.3. EPR and ESR Spectroscopy

Electron paramagnetic resonance (EPR) and electron spin resonance (ESR) are defining features of all melanin, by which the presence of stable groups of organic free radicals could be detected, these free radicals result in characteristic EPR behavior, confirming the melanin feature [78]. However, the typical melanin spectrum that indicates the presence of free radicals falls between 3300 and 3500 gausses [64]. 

#### 6.3.4. Mass Spectrometry

Usually, the mass spectrometer is utilized to quantify known compounds, to identify an unknown compound, determination of the compound’s molecular weight, and identify the structural and chemical properties. Investigation of melanin with mass spectrometry coupled with fast atom bombardment, laser desorption/ionization, and matrix-assisted laser desorption/ionization mass spectrometry proved that the darker melanin types have higher molecular masses, the degree of polymerization possibly is positively correlated to higher darkness of the molecule [94]. 

#### 6.3.5. High-Performance Liquid Chromatography (HPLC)

Chemical or thermal degradation using drastic procedures that produce an extensive breakdown of the pigment, followed by the identification of the end products by HPLC or gas chromatography coupled with mass spectrometry, have been used to recognize the monomeric items [95]. Recently, an improved HPLC technique has been applied for the detection of the two major classes of melanin pigments (eumelanin and pheomelanin), in biological samples, the method proved to be simple, specific, and reproducible [96].

#### 6.3.6. X-ray Diffraction

Generally, X-ray diffraction has been used for the characterization of several natural and synthetic types of purified melanin. X-ray diffraction of melanin shows the lack of crystalline structure in the diffraction pattern i.e., no significant crystallinity in melanin could be detected. The X-ray diffraction pattern was used to classify various kinds of biological melanin from the diffraction pattern of synthetic melanin [97]. Another work on natural and synthetic melanin reported the presence of high resemblance in the scattering intensity profiles, signifying that both kinds of melanin may be essentially similar in local atomic arrangements [98].

#### 6.3.7. Nuclear Magnetic Resonance (NMR) Spectroscopy

Little is known about NMR spectroscopy of melanin pigment, this limitations of NMR in melanin identification may back to the presence of free radicals, molecular complexity, and the scarce solubility, causing significant restriction on obtaining structural information of melanin [78]. However, NMR can be used for the determination of some melanin structures, i.e., the key functional groups in melanin such as the ketone, alkene, ester, alkane, alcohol, and indole units [99].

## 7. Current Obstacles

Owing to its unique features, melanin is dominant in potential applications in diverse life forms, especially in the medical sciences, and this, consequently, urges researchers to find out alternative efficient microbes and procedures for boosting the biosynthesis process to minimize the chasm between production and demand.

Natural melanin, like those generated from cuttlefish and fungi, are insoluble in water, thus, require severe treatments e.g., boiling in strong alkali or using strong oxidants for converting them into water-soluble form are applied. The harsh use for solubilization of the insoluble melanin frequently destroys the natural pigments and limits their usage.

Synthetic soluble melanin can be manufactured enzymatically by converting melanin precursors into pigments [9]. Unfortunately, these precursors are costly, resulting in a higher cost of artificial melanin. Similarly, vegetable melanin is a different source of true or eumelanin and is also expensive. One more significant limitation in utilizing natural melanin in biotechnology has been the low return of and the related extraction difficulties, as that occurs when separating melanin under brutal conditions, boiling utilizing NaOH for example [69,89]. On the other side, microorganisms secrete bulky quantities of extracellular melanin in aqueous media and have remarkable potential in biotechnological applications in contrast to insoluble melanin. Attaining low-cost soluble natural melanin can essentially promote and speed up the utilization of melanin in medicine, cosmetics, and several other fields. 

So, trials are in progress on microbial strains that produce soluble pure melanin. However, the next proposals (approaches) could be mentioned in the way to achieve the target. It is important to note that, till now some of these proposals are rarely used and others are not applied yet.

## 8. Next Scenarios

### 8.1. Application of the Statistical Approach

Conventional methods of optimization of medium conditions, using one variable at-a-time, are laborious, boring, and generate conflicting yield as they disregard interface among the tested production factors. The recently emerging tools of statistical experimental designs for optimization of the biosynthesis conditions of melanin have overcome the limitation of the conventional methods. 

The statistical modeling approach starts with the screening of the significant factors among multiple tested factors concurrently. Plackett-Burman designs (PBD) are the most applied methods to signify the important factors, the nonsignificant factor(s) that shows a very low effect on response values (melanin for example) are omitted from further experiments, whereas the significant ones are selected for extra optimization tests. Following the screening design and the related results, the response surface methodology (RSM) approach is applied to the significant factors to explore the relationship between the tested variable and response (melanin production). RSM contains several fixable designs to meet a variety of experimental conditions such as the number and concentration of the tested factors, nature of the design space, and the number of trials used. Central composite design (CCD) and the Box-Behnken design are the two main common approaches for such optimization and maximization process. As could be seen this modeling approach requires only two steps, followed by validation of the overall process [15,100].

However, rare studies on using PBD and RSM for melanin biosynthesis were applied, mostly in the last decade. As an example, among 17 independent factors, PBD was conducted, and three significant factors affecting melanin production by *Streptomyces glaucescens* were selected to study and optimize their interaction using CCD, leading to a maximum melanin production of 310.650 μg/1 mL [4]. Similarly, the boost in the melanin biosynthesis by *Auricularia auricula* was studied, engaging PBD, and CCD approaches, under the obtained optimized circumstances, the melanin yield was 1.08 g/L contrasted to 306.52 mg/L at suboptimal conditions, indicating a 3.52-fold increase [101]. 

The two abovementioned examples concluded that the statistical approach develops a low-cost and fast fermentation process with enhances melanin biosynthesis. This, in turn, encourages the scientific community to broaden the use of such techniques in the next era.

### 8.2. Artificial Intelligence Approach

Artificial intelligence is the distinguished sign of the next age. The melanin production process could be undergone optimization of the factor controlling the biosynthesis process. The amount and rapid development of data in recent years have motivated scientists to ponder how to obtain valuable information from the large quantity of gathered data through handling and analysis. For the past couple of years, artificial intelligence (AI) approaches have been quickly evolved and applied practically in a wide range of industries and biotechnology. By the same token, microbial melanin production can be magnified using AI. AI behaves like the human brain through learning, solving problems, perception, understanding, reasoning, critical thinking, and awareness of surroundings [102]. Among the regular and significant AI strategies, artificial neural networks (ANN) have drowned the greatest consideration regarding the ability in dealing with huge information, plan their nonlinear connections, and give outcome expectations [103]. ANN has been broadly applied in various fields, this employment benefits from the great self-learning ability and precision of ANN in modeling a complex relationship between input (tested factors) and output data (target product) without accommodating complicated numerical equations [104]. 

The core principle of ANN is based on the human brain’s learning behavior. ANN utilizes technology solutions to mimic the structures and functions of the human neural system, where learning in the human brain requires changing neurons, and synaptic interactions to achieve a specific target [102]. In this sense, ANN is amongst the scientific modeling tactics that employ neural network constructions to mimic the corresponding physical systems. The idea of ANN is that it provides a good nonlinear mapping capability, allowing to solve the challenge of mapping data from one set to another [105]. 

Anyhow, two main classes of ANN were identified, feedforward and feedback neural networks, both of which contain different frameworks, reflecting the wide elasticity and applicability in solving a variety of problems, the two classes simulate the synaptic performance among brain neurons by utilizing a huge number of nonlinear, and parallel processors to attain the function of learning [13]. The learning process of ANN forms mainly a relationship between input and output data, rather than focusing on the detailed interactions of physical and/or chemical conditions, and that is why ANN exactly generates the nonlinear mapping between inputs (tested variables) and outputs (target molecule e.g., melanin) to achieve the maximum prediction performance, usually, better than other modeling approaches like RSM [105]. 

ANNs are, in this sense, black-box models with great simulation accuracy, and they are often selected when the system’s intrinsic physical mechanics are highly complex or insignificant. With these explanations, ANNs could be applied in the modeling of the previous and current data, as well as, planning the future investigation on melanin biosynthesis on AI-base. This will expect to solve many melanin-related issues. To the authors’ knowledge, there is no literature dealing with the application of ANN during the optimization of the production process of melanin.

### 8.3. Economy Substrate Approach

Employing diverse aromatic precursors in the melanin production medium can generate various types of pure melanin. Despite this advantage, the relatively high cost of employing pure melanin precursors is a significant drawback [14]. Consequently, more efforts are required for the economization of melanin production by using agro-industrial byproducts as an eco-friendly alternative. Studies should be more directed towards semi-industrial production of melanin using an uncomplicated cultivation process by avoiding the utilization of expensive chemicals e.g., purified tyrosinase, complicated methods, and the cumbersome withdrawal procedure of melanin polymers. Future investigations need to be more focused on the pharmacological activity of melanin pigments which would be very helpful in designing a novel strategy for the management of some diseases like cancer.

Another rarely used approach is the utilization of agro-industrial residues as a fermentation substrate. Besides being readily available, the residues of agro-industrial biomass, as fermentation substrates, make them an economically reasonable choice for microbial melanin biosynthesis. In this connection, fruit residues extract, as a carbon source for bacterial fermentation, is an example of an inexpensive culture medium, which was validated in *Bacillus safensis* without additional nitrogen source, which was, apparently, 15% greater than the average yield, being 6.96 g/L [47].

### 8.4. Recombinant Microbes’ Approach

The experimental procedures, known as genetic engineering methods, utilize the alteration of microorganisms’ genetic components to increase biosynthesis or create new certain compounds. It is now feasible to genetically modify a wide range of microbes, and the number is rising all the time. Manipulation of culture conditions, in conjunction with recombinant technology, has been proven in studies to enhance melanin yield in large-scale manufacturing [14,15].

Therefore, attempts are being made for obtaining microbial strains that produce pure soluble melanin. For example, recombinant microorganisms, like *E. coli*, have been used for the biosynthesis of soluble melanin [9,106]. Another example, *Streptomyces glaucescens* successfully produced a water-soluble and optically clear, dark solution of melanin in a relatively short (2 days) incubation period [4,9], suggesting amenability to a wide variety of uses.

#### 8.4.1. Expression of Genes Encoding Tyrosinases

Gene cloning or transferring is an old-new procedure. The organism is modified to express genes encoding tyrosinases received from another organism. An early study reported that the recombinant *E. coli* exhibited eumelanin biosynthesis from L-tyrosine on agar-plates, and liquid medium after transferring the *mel* gene from *Streptomyces antibioticus* [107]. 

Expression of genes encoding tyrosinases can be performed through induction of the microorganisms that contain the melanin gene. In an early model, *Bacillus thuringiensis* was displayed to deliver melanin when cultured for several hours with L-tyrosine at 42 °C [62]. These outcomes indicate that the bacteria should be induced during the fermentation protocol since it contains a gene encoding a tyrosinase in its genome that was induced by the substrate (tyrosine) during the microbial development in the cultivation medium. Furthermore, the concentration of copper, which functions as a cofactor of tyrosinases and laccases, is necessary for the biosynthesis of both DHN- and DOPA-melanin, and so, copper can modulate the melanin synthesis pathway. As a result, copper can change melanin synthesis by controlling the expression of both enzymes. With the same given, cancellation of the copper-transporting ATPase gene was found to control the melanization process in *Botrytis cinerea* [108]. 

Another, physical conditions (e.g., pH, temperature, incubation periods) and specific media components can control gene expression, hence the biosynthesis process. Therefore, these conditions are usually altered according to the individual melanogenic strains [15]. Modulation of the growth conditions can positively or negatively change the genes’ expression and activate or stop the cryptic biosynthetic pathways of the pigments. Accordingly, genetic modification is strongly correlated to fermentation conditions, both are salient features for the enhancement of melanin biosynthesis.

In parallel, the symbiotic bacterium, *Rhizobium etli*, can fix nitrogen by forming symbiotic nodules in the root of *Phaseolus vulgaris*. The gene encoding tyrosinase (*mel*A) has been detected on the symbiotic plasmid. The *mel*A was cloned, in the expression vector pTrc99A, under control of the strong promoter (Trc), then the resultant plasmid (pTrc*mel*A) was transformed in *E. coli* strain, which bio-synthesized eumelanin in the L-tyrosine-containing medium Interestingly when compared to the original wild strain, the recombinant bacterium exhibited extensively higher survival rate [15,109].

#### 8.4.2. Random Mutagenesis

For gaining a novel melanogenic strain, another route, of recombinant microbes, can be gone through, which is random mutagenesis. To discover more about the role of melanogenesis genes, *Pseudomonas putida* strain grown in a medium containing L-tyrosine was used as a model for transposon mutagenesis study. This resultant mutant had high melanin biosynthesis ability, being a 6-fold increase, and superior resistance to UV light, and H_2_O_2_ than the wild strain. Genetic investigation revealed that the transposon mutagenesis method disrupted a gene encoding homogentisic acid 1,2-dioxygenase that converts homogentisic acid (originated from the L-tyrosine biosynthetic pathway) into 4-maleylacetoacetate as part of a catalytic pathway. This indicates that homogentisic acid is the precursor of allomelanin in this mutant strain [15].

A significant benefit of melanogenic species is the visual ease with which mutants can be identified, and more, the generated novel genes involved in the melanogenesis process could be discovered. A random mutagenesis is a straightforward approach for strain improvement, but, on the other hand, it is confined to species that already can produce melanin. Furthermore, the genetic alterations induced by random mutagenesis might be unstable, causing the strain to return to a low producer phenotype. A solution to this issue can be built on genome sequencing, to obtain enough information about the type of mutation as well as the genes and pathways involved in the observed new phenotype. This can help in recovering the identified mutations if lost and helps also in the separation of the genetic alterations that are responsible for the newly developed phenotype from those resulting from genetic instability [14].

#### 8.4.3. Metabolic Engineering

One of the drawbacks that appear in the employment of complex media components, is the lowered purity of melanin. Therefore, the efforts for the purification of melanin become a determinant issue on the industry level. Metabolic engineering proposes the application of simpler carbon substrates, during production, to induce the biosynthesis of both melanin and its precursor by the microorganism. For instance, a metabolic engineering method was applied to induce *E. coli* strain to synthesize the eumelanin precursor; L-tyrosine, from glucose. To direct the carbon flow from central metabolism into the common aromatic and the L-tyrosine biosynthetic pathways, feedback inhibition resistant versions of key enzymes were expressed in an engineered strain lacking the sugar phosphotransferase system and TyrR repressor. The expressed tyrosinase consumed intracellular L-tyrosine, causing growth impairment. To avoid this issue, a two-phase production process was devised, where tyrosinase activity was controlled by the delayed addition of the cofactor Cu. Following this procedure, melanin was produced with a simple carbon source as glucose. This strain had the potential for synthesizing eumelanin from glucose, with the aid of metabolic engineering, the strain was metabolically modified to overexpress the genes responsible for carbon flow to the L-tyrosine biosynthetic pathway [15,110], which is a precursor for melanin biosynthesis. The process reduced the production cost compared with employing L-tyrosine as raw material.

### 8.5. Green Nano-Melanin’s Approach

The green synthesis of biomolecules, such as melanin, is based on green biomaterials obtained from nature. In comparison to other synthetic melanin polymers, melanin from biological origin has been demonstrated to be very promising and growing in the research community among distinct research areas, such as biomedicine [16].

The current approach applies melanin nanoparticles (NPs) in the biomedical field, to which promising additional capabilities have been attributed compared to the natural form. NPs are proving to be a viable option for the development of novel agents, such as drugs. Unlike microparticles, NPs have a larger surface area and are capable of surface modification, giving them higher selectivity and specificity for a particular target [111]. In medicine, for example, melanin nanocarrier is not applied only as a diagnostic tool but also photothermal therapy, and controlled drug release through chemotherapy, this double action is known as theranostics [16]. What is more, through surface modification of nano-melanin, it is possible to add certain specific molecules to target certain kinds of cells, (tumor tissue or bacteria, for example). Melanin NPs are also able to enhance the drug loading capacity and to stimulate a controlled drug delivery, since, once in the vascular system, these NPs structures can enter cells by receptor-mediated transcytosis, or endocytosis, which rises the delivery of the NPs into the cells. Besides, melanin NPs can be expelled through the common organs, such as liver and kidney pathways, easier than higher-sized particles, presenting robust biocompatibility, and lower toxic effects emerging from the long-term accumulation in organs [16,28].

Consequently, microbial melanin NPs is a virgin area of study, to the best of our knowledge, no previous work was performed on the conversion of microbial melanin into nano form using green chemistry, or through a microbial factory, encouraging extensive studies in this biotechnological approach.

## 9. Drawbacks and Limitations of Melanin

Various drugs and other chemicals, such as organic amines, metals, etc., are bound to melanin and retained in pigmented tissues for long periods. The physiological significance of the binding is not evident, but it has been suggested that melanin protects the pigmented cells and adjacent tissues by adsorbing potentially harmful substances, which then are slowly released in nontoxic concentrations. Long-term exposure, on the other hand, may build up high levels of noxious chemicals, stored on the melanin, which ultimately may cause degeneration in the melanin-containing cells, and secondary lesions in surrounding tissues. In the eye, e.g., and in the inner ear, the pigmented cells are located close to the receptor cells, and melanin binding may be an important factor in the development of some ocular and inner ear lesions. In the brain, neuromelanin is present in nerve cells in the extrapyramidal system, and the melanin affinity of certain neurotoxic agents may be involved in the development of parkinsonism, and possibly tardive dyskinesia. In recent years, various carcinogenic compounds have been found to accumulate selectively in the pigment cells of experimental animals, and there are many indications of a connection between the melanin affinity of these agents and the induction of malignant melanoma [112]. The mechanism behind the development of lesions in the pigmented cells is probably a combination of selective retention, due to melanin binding, and toxicity.

The insolubility of melanin is another limitation for its absorption by cells as well as its applications. Water-soluble melanin, on the other side, is more efficient in various biotechnological attributes, including the medical application e.g., the antiviral activity of soluble melanin against human immunodeficiency virus [21,22]. So, it is critical for melanin to be water-soluble. To extend the applicability of melanin, various scenarios of research have focused on the solubilization of melanin using various methods, in this respect, several technologies have been used to obtain soluble melanin from insoluble ones such as squid ink [113]. The solubilization and/or the degradation of melanin may be related to the melanin structure and the technology used.

More melanin is not always better. In addition to its photoprotective feature, melanin can be toxic to cells and cause skin cancer. UV exposure energizes an electron in melanin, producing reactive oxygen compounds that can lead to a break in a single strand of DNA, and pheomelanin can generate hydrogen peroxide which may cause carcinogenic mutations. Moreover, DNA damage continued in human melanocyte cells even after hours of exposure to UV, concluding that melanin can cause skin cancer [114].

Insoluble melanin requires severe treatments such as chemicals, enzymes, boiling in strong alkali, or the use of strong oxidants for making them water-soluble, fortunately, the process can hardly split melanin monomers but is often associated with melanin damages [90]. For example, the application of ultrasound during the solubilization of melanin increased the propagation of ultrasound pressure waves and resulting cavitation phenomenon [115], concluding that the solubility may not be related to the degradation of melanin structure. Therefore, some supplementary means for the ultrasound degradation process, such as combining the use of ultrasound and enzyme, or ultrasound under hydrogen peroxide and alkaline conditions, were used to accelerate the degradation of the polymer, and keep its undegraded form [113,116]. In this respect, a novel method using ultrasound degradation under alkaline conditions (0.5 M NaOH) for preparing water-soluble squid ink melanin fractions, this combined method minimized the structural variation of the resulting soluble melanin fractions [112]. 

To avoid the damage of melanin fractions by the severe treatments, another scenario was proposed, which is the application of microorganisms in the production of the required type of melanin; soluble, insoluble, or both. *Streptomyces lusitanus* DMZ-3 is an example of a good producer of both kinds of melanin [117].

## 10. Conclusions

Melanogenesis is a vital process for living organisms. The natural complex polymer pigment, melanin, is abundantly detected in a wide array of higher organisms, and microorganisms, as well, where such pigments play vital and multi-function roles. Therefore, the current review spotted some light on natural melanin with special referencing to the microbial source. The current application, characterization, and drawbacks of melanin are explored. The next scenarios such as artificial intelligence, recombinant microbes, and green nano-melanin’s synthesis are the next approaches for melanin synthesis. Finally, future perspectives must devote more attention to the abovementioned topics for the economization of melanin production by using agro-industrial byproducts as an eco-friendly alternative. Studies should be more directed towards semi-industrial production of melanin utilizing a simple microbial cultivation process, consequently, avoiding the use of purified tyrosinase, expensive chemical methods, and the cumbersome extraction of the polymer from the plant, and animal tissues. Future investigations need to be more focused on the pharmacological activity of melanin pigment and its NPs, which would be very helpful in designing a novel strategy for the management of some diseases like cancer.

## Figures and Tables

**Table 1:** List of some bacteria and fungi that produce microbial melanin pigments.

Group	Microorganism	Objective	Main Finding	Reference
Bacteria	*Bacillus cereus*	Detection of melanin produced by a wild-type strain of *Bacillus cereus*	Melanin produced by the wild bacterium was firstly identified and its UV protection to insecticidal proteins was approved	Zhang et al. [61]
	*Bacillus thuringiensis*	Melanin pigment formation in high temperature	The bacterial cell was able to produce melanin in the presence of L-tyrosine at elevated temperature (42 °C).	Ruan et al. [62]
	*Burkholderia cepacia*	Attenuation of monocyte respiratory burst activity	Melanin-producing *B. cepacia* may derive protection from the free-radical-scavenging properties of this pigment.	Zughaier et al. [63]
	*Klebsiella* sp. GSK	Purification and physicochemical characterization of melanin pigment	A bacterium capable of producing a high amount of melanin from L-tyrosine within 3 days of incubation.	Sajjan et al. [64]
	*Pseudomonas stutzeri*	Melanin production from *Pseudomonas stutzeri* isolated from red seaweed *Hypnea musciformis*	The marine *Pseudomonas stutzeri* strain produces significant amounts of melanin of about 6·7 g l−1 without L-tyrosine supplementation in the sea-water production medium.	Ganesh Kumar et al. [65]
	*Pseudomonas maltophilia* *Aeromonas media*	Novel strain producing high levels of DOPA-melanin and assessment of the photoprotective role of the melanin	A novel melanin-producing bacterium was isolated. The melanin produced by this strain offers effective photoprotection of a commercial bioinsecticide against UV and solar radiation.	Wan et al. [44]
	*Stenotrophomonas maltophilia*	Isolation of *Stenotrophomonas maltophilia* from clinical samples and production of melanin pigment	*Stenotrophomonas maltophilia* was reported as a possible melanin source in the clinical environment, and the isolated bacteria showed production of melanin pigment with rates of strong, moderate, weak, and lack of pigment.	Amoli et al. [66]
Actinomycetes	*Nocardiopsis dassonvillei*	Extract bioactive melanin pigment from marine actinobacteria, which is not a widespread occurrence.	First report on the production and characterization of melanin from marine by *Nocardiopsis dassonvillei*.	Kamarudheen et al. [67]
	*Streptomyces cyaneus*	Optimization of medium conditions using response surface methodology for melanin production by *Streptomyces cyaneus* and synthesis of copper oxide nanoparticles using gamma radiation	The unprecedented achievement was realized for melanin pigment production, (9.898 mg/mL) was obtained by optimized culture condition. Also, 2.0% faba bean’s seed peel maximized melanin (9.953 mg/mL) and hence super-yield (11.113 mg/mL) was produced by a stimulus from gamma irradiation (2.5 kGy).	El-Batal et al. [68]
	*Streptomyces* spp.	Separation, identification, and analysis of melanin production in Streptomyces	The study reveals that the method of testing melanin production by L-tyrosine or L-dopa as a substrate may be a good criterion for the identification and classification of Streptomyces.	Dastager et al. [69]
Yeasts	*Cryptococcus neoformans*	melanin role in *Cryptococcus neoformans* virulence mechanism of action	Melanin appears to contribute to virulence by protecting fungal cells against attack by immune effector cells.	Wang et al. [59]
	*Yarrowia lipolytica*	Characterization of a nontoxic pyomelanin pigment produced by the yeast *Yarrowia lipolytica*	The ability of the yeast *Yarrowia lipolytica* W29 to produce high yield (0.5 mg/mL) extracellular melanin was reported in a culture medium supplemented with L-tyrosine. The purified pigment was found embedded with antioxidant properties	Ben Tahar et al. [70]
	*Hortaea werneckii*	Melanin is crucial for *Hortaea werneckii* growth in a hypersaline environment	Melanin has an important role in the ability of the black fungus *Hortaea werneckii* to survive in hypersaline environments.	Kejžar et al. [71]
Fungi	*Amorphotheca resinae*	Production and characterization of melanin pigments derived from *Amorphotheca resinae*	*Amorphotheca resinae* produced melanin in the peptone yeast extract glucose broth, reaching up 4.5 g/L within 14 days. The structural properties of melanin are similar to eumelanin.	Oh et al. [60]
	*Aspergillus bridgeri*	Physicochemical characterization and antioxidant activity of melanin	The extracellular pigment was alkali-soluble, acid-resistant, and insoluble in organic solvents and water. The pigment was precipitated and characterized and showed good free radical scavenging activity.	Kumar et al. [72]
	*Aspergillus fumigatus*	Production of pyomelanin via the tyrosine degradation pathway	The fungus was able to produce pyomelanin, by a different pathway, starting from L-tyrosine. Proteome analysis indicated that the l-tyrosine degradation enzymes are synthesized when the fungus is grown with L-tyrosine in the medium. Homogentisic acid is the major intermediate, and the L-tyrosine degradation pathway leading to pyomelanin is similar to that in humans leading to alkaptomelanin.	Schmaler-Ripcke et al. [73]
	*Aspergillus nidulans*	Characterization of fungal melanin pigment	The characterization of this pigment indicated the presence of indolic units, which were also found in synthetic DOPA-melanin. The analyses of the elemental composition showed that the pigment extracted from these mutants has a high percentage of nitrogen and, therefore, it cannot be DHN-melanin, which presents only a trace of nitrogen. Taken together, the results obtained in this study indicate that melanin produced by these mutants is DOPA type, representing the first report on the characterization of this type of melanin in *A. nidulans*.	Gonçalves et al. [74]
	*Auricularia auricula*	Auricularia auricula melanin and its molecular structure	The nutritional control was very important to promote melanin production, deficiency of tyrosine in the medium led to weak secretion of melanin. Meanwhile, the molecular and structural formulae concluded the presence of eumelanin	Sun et al. [75]
	*Cryomyces antarcticus*	Multidisciplinary characterization of melanin pigments from the black fungus *Cryomyces antarcticus*	The fungus possesses the ability to produce both 1,8-dihydroxynaphthalene (DHN) and L 3–4 dihydroxyphenylalanine (L-DOPA) melanins, opening interesting scenarios for the protective role against radiation.	Pacelli et al. [76]
	*Phyllosticta capitalensis*	Characterization of fungal endophyte melanin	First report of *Phyllosticta* melanin. Melanin in the hyphae of *P. capitalensis* may be responsible for the success of this fungus as a cosmopolitan endophyte since melanin is known to enhance the survival capability of fungi in stressful environments.	Suryanarayanan et al. [77]
	*Pleurotus cystidiosus*	Isolation and characterization of melanin pigment from *Pleurotus cystidiosus*	First report on isolation and characterization of melanin obtained from *Pleurotus cystidiosus* var. *formosensis*. The black pigment was confirmed as melanin based on UV, IR, and EPR spectra	Selvakumar et al. [78]
	*Spissiomyces endophytica*	Characterization and production of melanin by an endophytic fungus	The pigment was extracted, purified, and identified from the dried fungal biomass. The highest fungal pigment yield was observed in glucose yeast extract peptone medium at an initial pH value of 6.0 and 25 °C over three weeks of cultivation, representing the first report on the production and characterization of melanin obtained from the genus *Spissiomyces*.	Suwannarach et al. [57]

## Data Availability

All data were reported in the review.
